# Divergent Barmah Forest Virus from Papua New Guinea

**DOI:** 10.3201/eid2512.191070

**Published:** 2019-12

**Authors:** Leon Caly, Paul F. Horwood, Dhanasekaran Vijaykrishna, Stacey Lynch, Andrew R. Greenhill, William Pomat, Glennis Rai, Debbie Kisa, Grace Bande, Julian Druce, Mohammad Y. Abdad

**Affiliations:** Victorian Infectious Diseases Reference Laboratory of Royal Melbourne Hospital at the Peter Doherty Institute for Infection and Immunity, Melbourne, Victoria, Australia (L. Caly, J. Druce);; James Cook University, Townsville, Queensland, Australia (P.F. Horwood);; Monash University, Clayton, Victoria, Australia (D. Vijaykrishna);; AgriBio Centre for AgriBioscience, Bundoora, Victoria, Australia (S. Lynch);; Federation University Australia, Gippsland, Victoria, Australia (A.R. Greenhill);; Papua New Guinea Institute of Medical Research, Goroka, Papua New Guinea (W. Pomat, G. Rai, D. Kisa);; Divine Word University, Madang, Papua New Guinea (G. Bande);; National Centre for Infectious Diseases, Singapore (M.Y. Abdad)

**Keywords:** Barmah Forest virus, viruses, arbovirus, divergent strain, divergence, genomes, PCR, whole-genome sequencing, Papua New Guinea, Australia

## Abstract

We report a case of Barmah Forest virus infection in a child from Central Province, Papua New Guinea, who had no previous travel history. Genomic characterization of the virus showed divergent origin compared with viruses previously detected, supporting the hypothesis that the range of Barmah Forest virus extends beyond Australia.

Barmah Forest virus (BFV) is an arbovirus that is pathogenic to humans and is traditionally considered to be endemic only to Australia (*1*). BFV is a member of the Semliki Forest virus complex of the family *Togaviridae* (genus *Alphavirus*) that comprises several human arboviruses, including Ross River virus (RRV), Sindbis virus, and chikungunya virus. BFV was first isolated in 1974 from *Culex annulirostris* mosquitoes collected in the Barmah Forest within the state of Victoria and simultaneously from mosquitoes collected in southwest Queensland, Australia (*2*). Since then, it has been isolated in numerous other mosquitoes, including the coastal species *Aedes vigilax* (New South Wales) and *Ae. camptorhynchus* (Victoria), found in salt marshes and from the midge *Culicoides marksi* in Northern Territory (*3**–**5*). Subsequently, BFV has been detected in humans in most parts of mainland Australia, and serologic surveys have shown that this virus causes widespread infection (*6**–**8*).

BFV is transmitted to humans through bite from an infected mosquito, resulting in a mild disease and symptoms similar to those of RRV infection, including rash, fever, muscle tenderness, and polyarthralgia. Although the fever will generally pass within a week, muscle and joint pain may persist for >6 months (*9*), making BFV an infection of public concern. We report a case of infection with BFV in a child in Papua New Guinea.

## The Study

In April 2014, a boy (5 years, 11 months of age) who had no history of international travel came to an outpatient health clinic in a coastal village northwest of Port Moresby, Central Province, Papua New Guinea, because of an undifferentiated fever. Rash, muscle pain, and polyarthralgia were not noted at that time. Blood samples (containing EDTA anticoagulant) were collected as part of ongoing febrile illness surveillance and transferred to the Port Moresby laboratory of the Papua New Guinea Institute of Medical Research, where extraction of nucleic acids was performed.

We screened eluates by using a real-time reverse transcription PCR (RT-PCR) for a range of pathogens known to cause febrile illnesses, including BFV, chikungunya virus, dengue virus, Japanese encephalitis virus, RRV, West Nile (Kunjin) virus, Zika virus, *Orientia tsutsugamushi*, *Leptospira* sp., and *Rickettsia* sp. All test results were negative, except for a BFV TaqMan RT-PCR, which showed a positive result.

We isolated BFV by inoculating 100 μL of patient serum onto cultured Vero cells (strain PNG_BFV) and extracting and assessing the nucleic acid content of the harvested cell culture material by using a BFV-specific real-time RT-PCR. The result was positive, suggesting viral replication in culture and confirming the presence of BFV within the specimen of the patient.

We extracted RNA from the isolate material, prepared an RNASeq library by using the Scriptseq Version 2 Kit (http://www.epibio.com), and subjected this library to whole-genome sequencing by using the MiSeq System (https://www.illumina.com). We obtained 32 million paired-end reads and mapped them to the only available full-length (11,488-nt) BFV reference genome sequence (RefSeq accession no. NC_001786.1, strain ID BH2193) (*10*), which resulted in a complete PNG_BFV genome (GenBank accession no. MN115377) of 11,480 nt.

**Figure 1:**
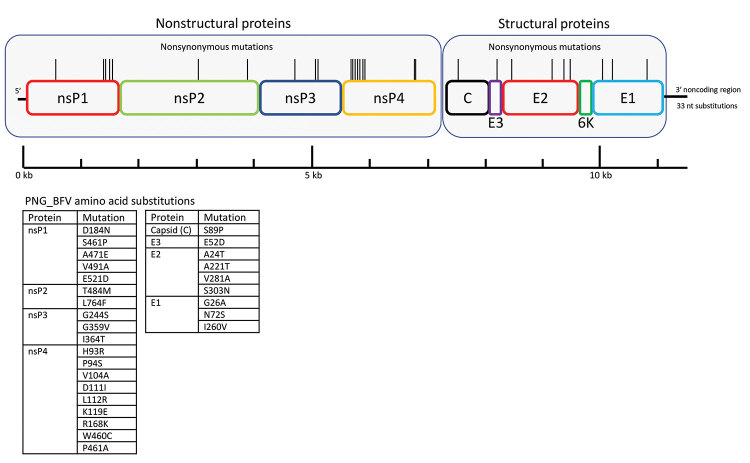
Schematic representation of the BFV genome showing location of amino acid differences between the PNG_BFV (MN115377) isolate from a child in Papua New Guinea and prototype strain BH2193 (RefSeq accession no. NC_001786.1). Amino acid substitutions in the PNG_BFV genome are shown in nonstructural proteins nsP1–4 (n = 19) and structural proteins C, E1–3, and 6K (n = 9) and listed below the schematic. BFV, Barmah Forest virus; C, capsid; E, envelope; nsP, nonstructural protein; PNG, Papua New Guinea.

Comparison of PNG_BFV with the reference genome showed the presence of 343 nt differences, which constitutes a 2.98% pairwise difference between PNG_BFV and prototype strain BH2193 (Table). Most changes were single-nucleotide polymorphisms, although these changes included multiple-nucleotide substitutions, and insertions and deletions (indels). A large number of these changes (219 nt) were found in the nonstructural polyprotein coding region 1–4, of which 23 were nonsynonymous, resulting in 19 aa changes (Figure 1).

**Table:** Synonymous and nonsynonymous differences between Barmah Forest virus isolate PNG_BFV from a child in Papua New Guinea and prototype strain BH2193*

Genome region	nsP1–4	Structural	3′
Synonymous	196	81	–
Nonsynonymous	23	10	–
Total	219	91	33

*Prototype strain, RefSeq accession no. NC_001786.1. nsP, nonstructural protein; –, none.

In addition, 91 nt changes occurred within the structural polyprotein coding region, 10 of which were nonsynonymous, resulting in 9 aa changes (Figure 1). We also observed an additional 33 nt substitutions in the 3′ noncoding end of the genome. The biologic context of these amino acid substitutions and their effects on virus pathogenicity, infectivity, and antigenicity remains to be determined and will be explored in further studies.

To determine the evolutionary relationship of the PNG_BFV strain with those detected in Australia, we estimated their phylogenetic relationships by using the maximum-likelihood method and the time to most recent common ancestor of each node by using Bayesian methods. We aligned the complete envelope (E2) sequences of all currently available BFV strains (n = 7) in the Virus Pathogen Resource database (https://www.viprbrc.org/brc/home.spg?decorator = vipr) and an isolate from Victoria (M4208_16/17) with the newly generated PNG_BFV envelope (E2) protein gene sequence of 1,263 nt.

**Figure 2:**
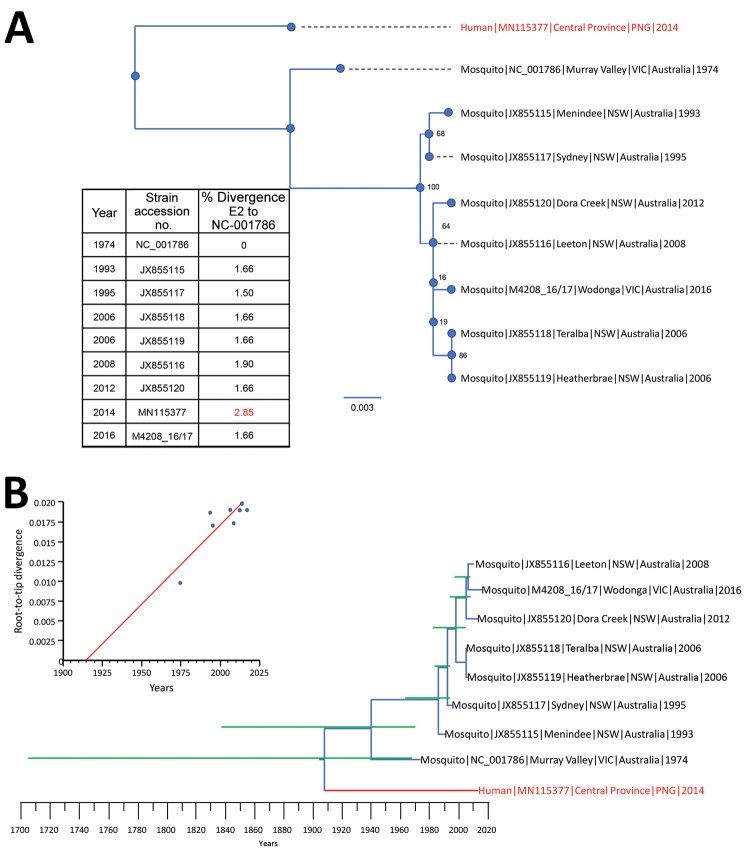
Phylogenetic relationships between 9 full-length (1,263 nt) Barmah Forest virus (BFV) envelope (E) protein genes. A) Maximum-likelihood phylogenetic tree constructed from 8 full-length Australia BFV E2 sequences (blue) and a BFV E2 sequences from an isolate from a child in Papua New Guinea (red) by using the best-fit nucleotide substitution model in IQ-Tree version 1.5 (*11*). Bootstrap values were estimated by using 1,000 replicates; percentages are indicated on branch nodes. Inset table shows E2 nucleotide divergence compared with that for prototype strain BH2193 (RefSeq accession no. NC_001786.1). Scale bar indicates nucleotide substitutions per site. B) Molecular clock analysis using the Bayesian Markov chain Monte Carlo method in BEAST (*12*) for 9 complete BFV E2 sequences (blue) spanning 1974–2016. Red indicates BFV from an isolate from a child in Papua New Guinea. Green lines indicate 95% CIs. Inset shows temporal analysis of root-to-tip linear regression by using TempEst version 1.5 (*13*). Slope, 1.98 × 10^−4^; X-intercept, 1914.2; correlation coefficient, 0.86; R^2^, 0.743; residual mean squared, 2.76 × 10^−6^. NSW, New South Wales; VIC, Victoria.

Phylogenetic analysis showed that PNG_BFV is divergent from known BFV strains from Australia, suggesting that the strain was not a recent introduction from Australia but has been evolving independently as a separate BFV clade for quite some time (Figure 2, panel A). Furthermore, we observed a greater nucleotide diversity of the E2 gene between the BFV reference strain (BH2139) and the Papua New Guinea strain (2.85%) than between all strains collected in Australia during 1974–2016 (1.50%–1.90%).

In an effort to determine the time of divergence of the Papua New Guinea strain from known strains from Australia, we first estimated a root-to-tip regression model to explore the temporal structure of the 8 BFV sequences by using Tempest version 1.5 (*13*). This estimation showed a slope of 1.98 × 10^−4^, which was comparable to nucleotide substitution rates of the surface proteins of RNA viruses (*14*) and also showed that this dataset contained adequate temporal signal for a robust estimation of substitution rates and divergence times (Figure 2, panel B).

Subsequently, we estimated the molecular clock for the final dataset of 8 complete E2 protein sequences with a sampling range of 1974–2016, under a stick clock model; a constant coalescent population size and the Hasegawa, Kishino, and Yano substitution model by using the Bayesian Markov chain Monte Carlo method in BEAST version 1.8 (*12*) (Figure 2, panel B). We determined the median root age to be during 1906 (95% CI 1703–1969) with a calculated mean nucleotide substitution rate of 1.7 × 10^−4^ (95% CI 5.4 × 10^–12^–3.3 × 10^−4^). The wide CIs suggest that sampling was inadequate to provide a precise estimate of the time of divergence and evolutionary rate, which would be greatly improved with access to additional BFV whole-genome sequences and full-length E2 gene sequences, which are currently not available for public access.

## Conclusions

We report a case of infection with BFV in a child who had no travel history from the Central Province of Papua New Guinea. BFV has been traditionally believed to be endemic only to Australia. Whole-genome sequencing, followed by phylogenetic analysis, showed that this strain was highly divergent from known strains from Australia. These findings placed the Papua New Guinea virus strain within its own clade and supported the hypothesis that the range of BFV extends beyond Australia. Molecular clock analysis indicates that the virus strains from Papua New Guinea and Australia probably diverged during or before the early 1900s, raising questions on the origins and the overall genetic diversity of BFV. On the basis of currently available data, the probable origins of these viruses, either from Australia or neighboring northern countries, such as Papua New Guinea, are inconclusive.

The timeline of divergence suggests that this divergence could have occurred by movement of humans, livestock, or mosquitoes from or to Australia during the early 1900s by trade routes or movement of troops during World War I. Increased mosquito surveillance and serosurveys of the population in Papua New Guinea is needed to determine the endemic nature of BFV, which is likely to extend beyond the single detection noted within the Central Province.
